# Tumor location as an indication for adjuvant radiotherapy in pT3N0 rectal cancer after surgery

**DOI:** 10.1186/s13014-019-1206-3

**Published:** 2019-01-16

**Authors:** Hai-hua Peng, Xin-hui Zhou, Tong-chong Zhou, Xing-sheng Qiu, Kai-yun You

**Affiliations:** 1 Department of Radiation Oncology, Affiliated Cancer Hospital & Institute of Guangzhou Medical University, Guangzhou, 510075 China; 2 Department of Radiation Oncology, SunYat-Sen Memorial Hospital, SunYat-Sen University, Guangzhou, China

**Keywords:** Rectal cancer, Chemotherapy, Radiotherapy, Tumor location

## Abstract

**Background:**

The optimal care for pT3N0 rectal cancer remains controversial. And whether tumor location can be used to guide the administration of adjuvant radiotherapy for pT3N0 rectal cancer is not fully confirmed. The current study was designed to identify the benefit of adjuvant radiotherapy for pT3N0 rectal cancer.

**Methods:**

We performed a retrospective study of 265 pT3N0 rectal cancer patients who were treated by surgery and adjuvant therapy from Mar. 2005 to Sept. 2015. All patients were divided into two groups according to receiving adjuvant radiotherapy or not. Overall survival (OS), disease-free survival (DFS) were compare between patients who did and did not receive adjuvant radiotherapy. Multivariate analysis was performed to explore clinical factors significantly associated with DFS, local recurrence-free survival (LRFS) and distant metastasis-free survival (DMFS).

**Results:**

For patients with lower tumor, DFS in adjuvant chemo-radiotherapy group was higher than that in adjuvant chemotherapy group. Besides, the rates of local recurrence and distant metastasis were found lower in patients who did receive adjuvant radiotherapy than those who did not. For patients with upper tumor, the 5-year OS and DFS were similar between groups of adjuvant chemotherapy and adjuvant chemo-radiotherapy. Multivariable analysis indicated both the CEA and tumor location were independent predictors of LRFS. And adjuvant radiotherapy predicted the DFS, LRFS and DMFS in lower rectal cancer patients.

**Conclusion:**

Tumor location can serve as an indication for the administration of adjuvant radiotherapy in pT3N0 rectal cancer patients.

## Introduction

The treatment of rectal cancer has rapidly evolved during the last 15 years with an increasing use of preoperative chemoradiotherapy for locally advanced disease, which has markedly improved the quality of life and survival of rectal cancer patients [1]. However, postoperative radiotherapy is also routinely used for patients who undergo surgery first and have a pathologic stage of pT3–4 or N+. Currently, radical surgery with the principle of total mesorectal excision (TME) is the standard operation form for a resectable rectal cancer, which has significantly decreased the local recurrence rate [2, 3]. Studies reported that the rates of local recurrence ranged from 4.1 to 6.5% in pT3N0 rectal patients who received TME surgery alone [2, 3]. Due to the low rate of local failure, the benefit of additional radiotherapy in this subset of rectal cancer is controversial [4, 5]. Several studies have reported that adjuvant radiotherapy may not improve the survival and local control rate in pT3N0 rectal cancer [6–8]. They suggested that post-operative adjuvant radiation may be unneeded and over-treatment for patients with pT3N0 stage. However, other researchers held different viewpoint of that the pT3N0 rectal cancer have an intermediate risk of recurrence, and more investigation was needed to confirm the real value of radiation therapy [9, 10]. In this study, our goal is to further explore the value of adjuvant therapy in pT3N0 rectal cancer patients. Besides, we try to find whether some routine clinical factors could be used to guide the administration of adjuvant radiotherapy.

## Materials and methods

### Patients selection

Data were extracted from a rectal cancer database that included all patients who underwent surgical treatment at Affiliated Cancer Hospital & Institute of Guangzhou Medical University from Mar. 2004 to Sept. 2015. The database included patient characteristics, operative findings, pathologic reports, adjuvant therapy, and follow-up data. The patient selection criteria were as the following: (1) without any preoperative therapy; (2) receiving TME surgery; (3) pathologically confirmed T3 N0 rectal adenocarcinoma; (4) receiving adjuvant therapy. When the reviewing of medical and pathologic records was completed, 280 patients with pT3N0M0 were initially selected,. Then 10 patients were lost in the follow up. And 5 cases with CRM positive were not as candidates. Finally, 265 patients were enrolled in the study.

### Clinical evaluation

Clinical staging was assessed according to ultrasound colonoscopy, computed tomography, magnetic resonance imaging (MRI), and chest radiography. Other examinations such as complete blood count and liver function test were also performed. Pre-treatment CEA levels were measured within 2 weeks before surgery. Although National Comprehensive Cancer Network (NCCN) guideline recommended preoperative chemoradiation for all locally advanced rectal cancer (cT3,4 or N+), this strategy has not been widely practiced until the year of 2007 in this hospital and surgery with adjuvant therapy is now still considered as an option for clinical stage T3N0M0. Besides, some patients who were clinically stage as T1-2 N0 received radical surgery, but with final pathology of pT3N0. The tumor location defined in current study was the distance from the anal verge (AV) to the primary tumor. And the distance was measured by ultrasound colonoscopy. Upper and lower rectal caner were defined as tumor located more than(≥7 cm) or within 7 cm(< 7 cm) from the anal verge [11, 12].

### MRI examination

Before surgery, all the patients were given MR(1.5-TeslaI) examination. The region from the apertura pelvis superior to the anus was scanned (supine position). The section thickness was 5 mm for the axial plane (1 mm interslice spacing) and 6 mm for the sagittal and coronal planes (1 mm interslice spacing). Usually, the T1-weighted images and T2-weighted images were obtained before the injection of contrast material, and the contrast-enhanced T1-weighted images were collected after the injection of contrast material. The tumor volume was calculated based on the pelvic MRI scanning images. And it was done by two observers, including one radiologists and a clinician.

### Treatment

In our study, all the patients were divided into two groups as follows. Group A (adjuvant chemotherapy group): the patients received adjuvant chemotherapy alone. Group B (adjuvant chemo-radiotherapy group): the patients received both adjuvant chemotherapy and adjuvant radiotherapy. Usually, when the patients have completed the treatment of surgery, they were given adjuvant concurrent chemo-radiation followed by adjuvant chemotherapy.

All the operations were performed according to the TME-principles by colorectal surgeons and the methods included low anterior resection, abdominoperineal resection and Hartmann. The four patients who underwent Hartmann’s procedure were due to the poor preoperative bowel preparations.

All of the patients received adjuvant chemotherapy or concurrent chemotherapy. The regimens of adjuvant chemotherapy included FOLFOX6 (oxaliplatin 85 mg/m2,d1 + leucovorin 400 mg/m2,d1+ 5-FU 400 mg/m2 iv d1, then 2400 mg/m2civ46-48 h), Xelox (oxaliplatin130mg/m2,d1 + capecitabine1000mg/m2bid, po, d1–14), and single agent capecitabine (capecitabine1250mg/m2bid, po, d1–14).when adjuvant chemotherapy was concurrent with radiotherapy. The regimens were as followings: 225 mg/m2 of 5-Fu over 24 h 5 days/week during radiotherapy or took Xeloda 825 mg/m2 twice daily 5 days per week during radiotherapy. There were 45 patients who were treated with concurrent chemotherapy of 5-FU, while the other 70 patients received capecitabine.

The technique of radiotherapy was based on three-dimensional conformal radiotherapy treatment planning system with 3 or 4 fields irradiation plan being used. The clinical target volume (CTV) included primary rectal tumor bed, peri-rectal tissues, pre-sacral lymph nodes, internal iliac lymph nodes and obturator lymph nodes. The superior border of the CTV was the bottom of L5, and the inferior border was the obturator formamen (Dixon) or 1.5 cm inferior of the metal sign (Mile’s),. The anterior border was the posterior margin of the bladder or uterus and the posterior border was the anterior margin of the sacrum. PTV is defined as CTV+ 8 mm. The prescription dose to the whole pelvis was 46–50 Gy in 23–25 fractions over 5 weeks.

### Pathologic evaluation

The pathology reports were reviewed for histologic differentiation, lymphovascular invasion, total number of lymph nodes retrieved, and the involvement of the circumferential resection margin (CRM). Additionally, CRM involvement in our study was defined as the microscopic presence of tumor cells from the outermost margin of the tumor to the proper mesorectal fascia or when the maximum distance between the tumor and proper rectal fascia was less than 1 mm.

### Follow-up

Follow-up examinations were performed every 3 months for the first 2 years after treatment and every 6 months thereafter. Evaluations included complete blood count, liver function test, CEA, CA19–9, physical examination and digital rectal examination for each visit. Chest radiography, CT scanning of the abdomen and pelvis, and colonoscopy were performed every 6 months after surgery. PET/CT were not regularly recommended in case of suspicious recurrence was detected. The follow-up of each patient was recorded in our database.

### Statistical analysis

All statistical analyses were performed by SPSS software, Version19.0. Categorical variables were analyzed using the chi-square test or Fisher’s exact test, and continuous variables were analyzed by the Student t test or Mann–Whitney U test. The Kaplan–Meier method was employed to compare DFS rates and OS rates. Univariate analyses of factors associated with DFS, LRFS, and DMFS were performed by the Kaplan–Meier method. Multivariate analysis was performed by Cox proportional hazards regression. Statistical significance was considered to be *p* < 0.05.

## Results

### Clinical characteristics

In all, there were 265 patients with pT3N0 on the final pathology enrolled in our study. Among them, 115 patients received postoperative adjuvant chemo-radiotherapy, while 150 patients just received chemotherapy. Compared to patients who received adjuvant chemotherapy, those who received chemo-radiotherapy were significantly younger (*P* = 0.041) and more likely to have a lower Hb level (*P* = 0.025). Other variables such as gender, location of tumor, tumor grade, type of surgery, tumor volume, number of retrieved lymph nodes were similar between two groups. Median follow-up for patients who did and did not receive adjuvant radiotherapy were 56.4 and 57.1 months, respectively. And there was also no difference between the two groups (Table 1). Besides, in the subgroup of patients with lower rectal cancer, the common baseline characteristics between patient who received adjuvant chemotherapy and adjuvant chemo-radiotherapy were all comparable (Table 2).

**Table 1:** Baseline characteristics for the whole group

Variable	Adjuvant chemotherapy group (*n* = 150)	Adjuvant chemo-radiotherapy group (*n* = 115)	*P* value
Age, year			0.041
median	57	55	
Gender			0.311
male	95	65	
female	55	50	
Hb g/L			0.025
< 128	67	68	
≥ 128	83	47	
CEA, ug/mL			0.083
< 5.00	75	70	
≥ 5.00	75	45	
Tumor Location from AV, cm			0.213
< 7.0	63	58	
≥ 7.0	87	57	
Tumor grade			0.421
G1	11	5	
G2	123	101	
G3	16	9	
Type of surgery			0.804
Mile’s	30	23	
Dixon	117	88	
Hartmann	3	4	
Tumor volume, cm^3^			0.083
< 19	68	65	
≥ 19	82	50	
Retrieved lymph nodes			0.174
< 14	69	63	
≥ 14	81	52	
Lymph vascular invasion			0.808
yes	11	7	
no	139	108	
Duration of adjuvant therapy, months			
median	5.3 (4.5–6.2)	5.0 (4.4–6.1)	0.485
Follow-up, months			0.872
median	57.1	56.4	

*Abbreviations*: *adjuvant-chemo* adjuvant chemotherapy, *Hb* hemoglobin, *CEA* carcinoembryonic antigen, *AV* anal verge, *tumor grade: G1* well differentiated, *G2* moderately differentiated, *G3* poorly differentiated

**Table 2:** Baseline characteristics for subgroup of patients with lower rectal cancer

Variable	Adjuvant chemotherapy group (*n* = 63)	Adjuvant chemo-radiotherapy group (*n* = 58)	*P* value
Age, year			0.053
median	56	55	
Gender			0.467
male	39	32	
female	24	26	
Hb g/L			0.202
< 128	30	35	
≥ 128	33	23	
CEA, ug/mL			0.203
< 5.00	29	34	
≥ 5.00	34	24	
Tumor grade			0.931
G1	4	3	
G2	52	50	
G3	7	5	
Type of surgery			0.833
Mile’s	27	26	
Dixon	35	30	
Hartmann	1	2	
Tumor volume, cm^3^			0.717
< 19	29	29	
≥ 19	34	29	
Retrieved lymph nodes			0.587
< 14	30	31	
≥ 14	33	27	
Lymph vascular invasion			0.719
yes	5	3	
no	58	55	
Duration of adjuvant therapy, months			0.523
median	5.3 (4.5–6.2)	5.1 (4.5–6.0)	

*Abbreviations*: *adjuvant-chemo* adjuvant chemotherapy, *Hb* hemoglobin, *CEA* carcinoembryonic antigen, *AV* anal verge, *tumor grade: G1* well differentiated, *G2* moderately differentiated, *G3* poorly differentiated

### Survival analysis for the whole group

**Fig. 1:**
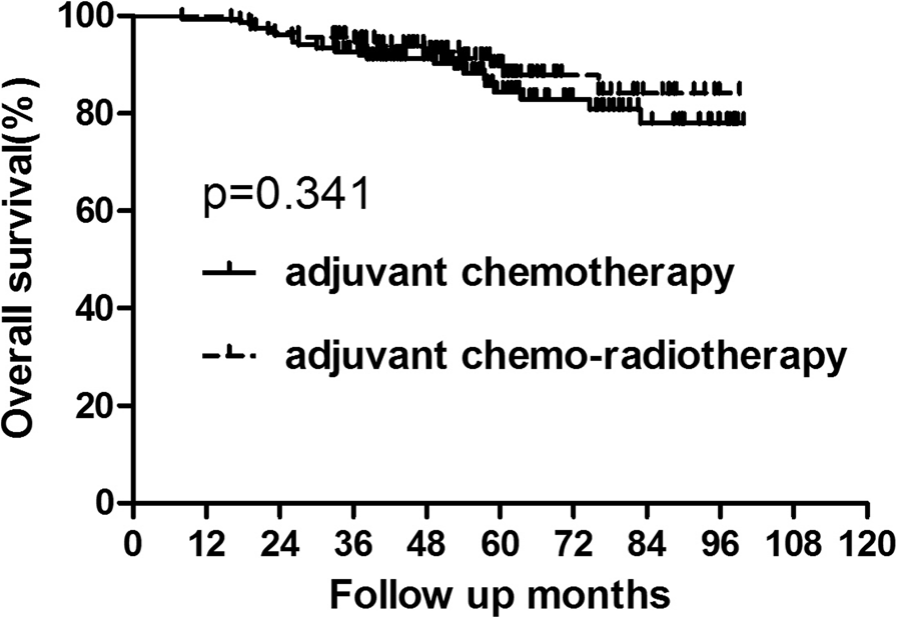
OS for the whole group patients stratified by treatment with adjuvant radiotherapy. No significant difference was found in OS between patients who did and did not receive adjuvant radiotherapy for whole group patients (*P* = 0.341)

**Fig. 2:**
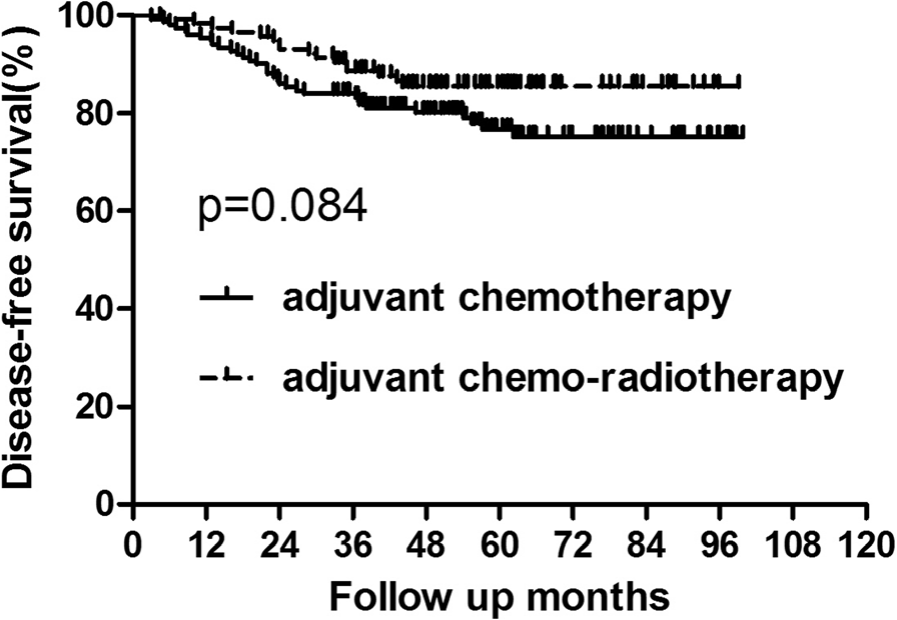
DFS for the whole group patients stratified by treatment with adjuvant radiotherapy. No significant difference was found in DFS between patients who did and did not receive adjuvant radiotherapy for whole group patients(*P* = 0.084)

For the whole group, 34 patients died of tumor recurrence during the follow up. And the 5-year OS in the chemotherapy and chemo-radiotherapy groups were 84.37 and 89.67%, respectively (Fig. 1, Table 3). No significant difference was showed between the two groups(*p* = 0.341). There were 49 patients who developed recurrence. Among patients who recurred, 24 patients were with local recurrence alone and 11 patients suffered from only distant metastasis. Additionally, 14 patients presented with both local and distant metastasis. The 5-year DFS were also similar between patients who did and did not receive adjuvant radiotherapy (76.70% vs 85.54%, *p* = 0.084) (Fig. 2, Table 3).

**Table 3:** OS and DFS for the whole group

Group	Adjuvant chemotherapy Group (*n* = 150)			Adjuvant chemo-radiotherapy Group (*n* = 115)		*P* value
	3-year	5-year		3-year	5-year	
OS	92.66%	84.37%		95.65%	89.67%	0.341
DFS	84.0%	76.70%		88.53%	85.54%	0.084

*Abbreviations*: *adjuvant-chemo* adjuvant chemotherapy, *DFS* disease-free survival, *OS* overall survival

### Subgroup analysis based on tumor location

**Fig. 3:**
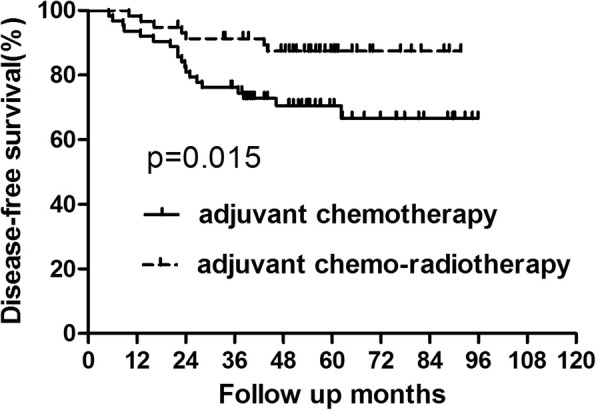
DFS for patients with lower rectal cancer stratified by treatment with adjuvant radiotherapy. Patients who received adjuvant radiotherapy acquired better DFS than those who did not (*P* = 0.015)

**Fig. 4:**
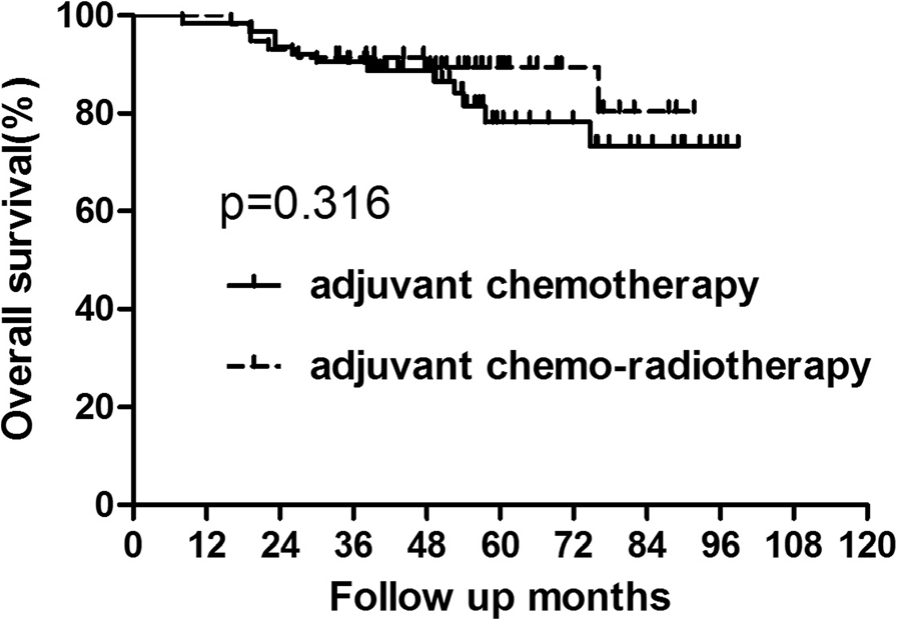
OS for patients with lower rectal cancer stratified by treatment with adjuvant radiotherapy. No significant difference was found in OS between patients who did and did not receive adjuvant radiotherapy for patients lower rectal cancer (*P* = 0.316)

For the patients with lower tumor, those undergoing adjuvant chemo-radiotherapy exhibited longer DFS than those undergoing adjuvant chemotherapy alone (*p* = 0.015) (Fig. 3, Table 4). However, there was no significant difference in OS between the two groups (*p* = 0.316)(Fig. 4, Table 4). Based on analysis of distant metastasis, the patients in the adjuvant chemo-radiotherapy group displayed a lower rate of distant metastasis than those in adjuvant-chemo group(*p* = 0.033). Besides, the rate of local recurrence was also found to be lower in patents who did receive adjuvant radiotherapy than those who did not (*p* = 0.032)(Table 5).

**Table 4:** OS and DFS for subgroup of patients with lower rectal cancer (tumor location< 7 cm from AV)

Group	Adjuvant chemotherapy Group (*n* = 63)			Adjuvant chemo-radiotherapy Group (*n* = 58)		*P* value
	3-year	5-year		3-year	5-year	
OS	90.48%	78.28%		91.38%	89.39%	0.316
DFS	76.19%	70.56%		91.32%	87.43%	0.015

*Abbreviations*: *adjuvant-chemo* adjuvant chemotherapy, *DFS* disease-free survival, *OS* overall survival, *AV* anal verge

**Table 5:** Recurrence Patterns for subgroup of patients with lower rectal cancer(tumor location< 7 cm from AV)

Group	Adjuvant chemotherapy Group (*n* = 63)			Adjuvant chemo-radiotherapy Group (*n* = 58)		*P* value
	3-year	5-year		3-year	5-year	
LR	8 (12.7%)	12 (19.0%)		4 (6.9%)	4 (6.9%)	0.032^a^
SM	10 (15.9%)	14 (22.2%)		3 (5.2%)	5 (8.6%)	0.033^a^

*Abbreviations*: *LR* local recurrence, *SM* systemic metastases, *AV* anal verge

^a^calculated by Kaplan–Meier method

**Fig. 5:**
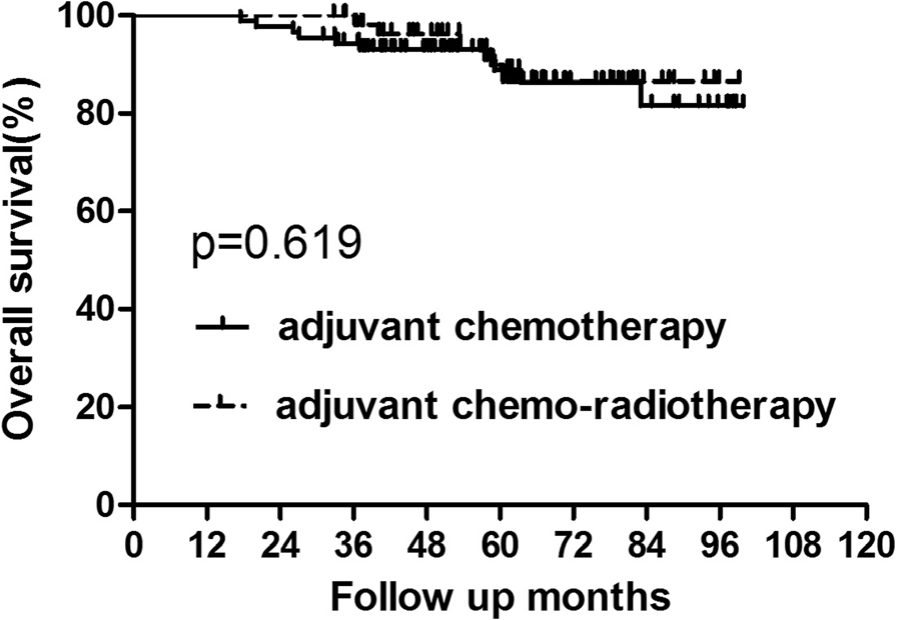
OS for patients with upper rectal cancer stratified by treatment with adjuvant radiotherapy. No significant difference was found in OS between patients who did and did not receive adjuvant radiotherapy for patients with upper rectal cancer (*P* = 0.619)

**Fig. 6:**
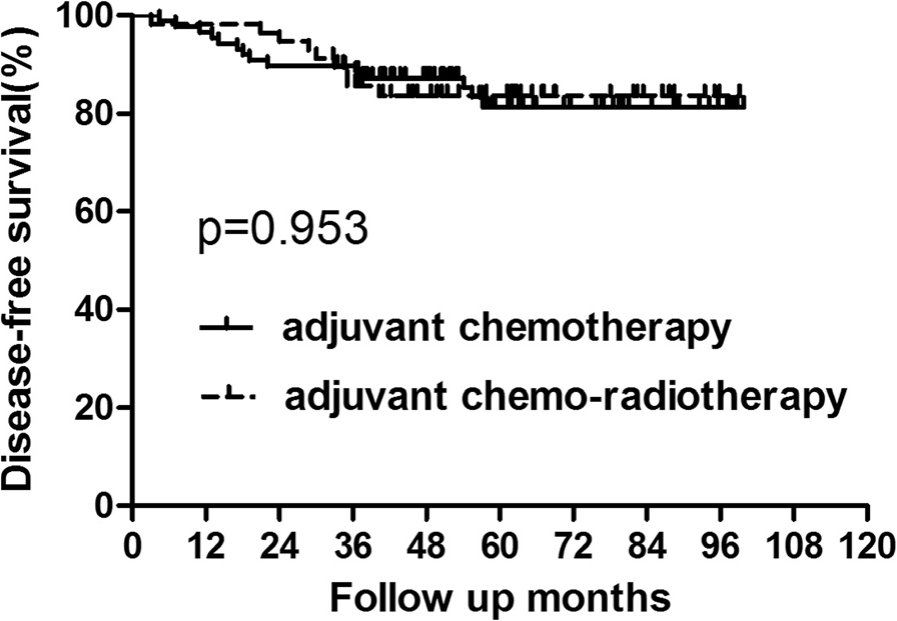
DFS for patients with upper rectal cancer stratified by treatment with adjuvant radiotherapy. No significant difference was found in DFS between patients who did and did not receive adjuvant radiotherapy for patients with upper rectal cancer(*P* = 0.953)

In patients with upper rectal cancer, 23 patients experienced recurrence and 15 patients died of tumour recurrence. Among patients who recurred, 4 cases displayed with only local recurrence and 15 patients exhibited only distant metastasis. And 4 patients developed both local and distant recurrence. In the chemotherapy and chemo-radiotherapy groups, the 5-year OS rates were 88.80 and 89.87%, respectively. And the 5-year DFS rates were 81.40 and 83.72%, respectively (Figs. 5 and 6, Table 6). No significant difference was detected in either OS (*p* = 0.619) or DFS (*p* = 0.953). Further analysis of the recurrence pattern revealed that there were no differences in both the local recurrence and distant metastasis rates between patients who did and did not receive adjuvant radiotherapy(*p* > 0.05).

**Table 6:** OS and DFS for subgroup of patients with upper rectal cancer(tumor location≥7 cm from AV)

Group	Adjuvant chemotherapy Group (*n* = 87)			Adjuvant chemo-radiotherapy Group (*n* = 57)		*P* value
	3-year	5-year		3-year	5-year	
OS	94.22%	88.80%		98.15%	89.87%	0.619
DFS	89.66%	81.40%		85.71%	83.72%	0.953

*Abbreviations*: *adjuvant-chemo* adjuvant chemotherapy, *DFS* disease-free survival, *OS* overall survival, *AV* anal verge

### Clinical predictors for DFS, LRFS and DMFS in the whole group

For the whole group, multivariable analysis showed that pre-treatment CEA level and retrieved lymph nodes were significantly associated with DFS. And for LRFS, results reveled that both the CEA level and tumor location were independent predictors of it. Besides, higher CEA level and fewer retrieved lymph nodes predicted poorer DMFS (Table 7).

**Table 7:** Multivariate Analyses of DFS, LRFS, and DMFS for the whole group

Variable	DFS		LRFS		DMFS	
	HR(95%CI)	*P* value	HR(95%CI)	*P* value	HR(95%CI)	*P* value
CEA, ug/mL						
< 5.00 vs ≥5.00	0.496 (0.280–0.879)	0.016	0.337 (0.145–0.781)	0.011	0.552 (0.0.316–0.902)	0.037
Tumor location, cm						
< 7.0 vs ≥7.0	NA		2.428 (1.046–5.635)	0.039	NA	
Number of retrieved lymph nodes						
< 14 vs ≥ 14	3.079 (1.652–5.736)	< 0.001	3.999 (1.574–10.165)	0.004	2.106 (1.077–4.118)	0.030

*Abbreviations*: *DFS* disease-free survival, *LRFS* local recurrence-free survival, *DMFS* distant metastasis-free survival, *NA* not available, *CI* confidence interval, *HR* hazard ratio

### Clinical predictors for DFS, LRFS and DMFS in patients with lower rectal cancer (tumor location< 7 cm from AV)

As for patients with lower rectal cancer, although tumor volume was not significantly associated with LRFS, it did predict both the DFS and DMFS. It was also found that the adjuvant radiotherapy predicted the DFS, LRFS and DMFS, meaning that the addition of radiotherapy could significantly improve the survival (Table 8).

**Table 8:** Multivariate Analyses of DFS, LRFS, and DMFS for subgroup patients with lower rectal cancer(tumor location< 7 cm from AV)

Variable	DFS		LRFS		DMFS	
	HR(95%CI)	*P* value	HR(95%CI)	*P* value	HR(95%CI)	*P* value
Tumor volume, cm^3^ < 18 vs ≥18	0.429 (0.183–0.906)	0.047	NA		0.217 (0.061–0.769)	0.018
Adjuvant radiotherapy yes vs no	0.358 (0.150–0.851)	0.020	0.314 (0.102–0.964)	0.043	0.345 (0.124–0.959)	0.041

*Abbreviations*: *DFS* disease-free survival, *LRFS* local recurrence-free survival, *DMFS* distant metastasis-free survival, *NA* not available, *CI* confidence interval, *HR* hazard ratio, *AV* anal verge

## Discussion

Our current study demonstrated that no significant differences were found in both OS and DFS in pT3N0 rectal cancer patients who received adjuvant chemo-radiotherapy compared to adjuvant chemotherapy. However, when subgroup analysis was performed, it surprisingly showed that adjuvant radiotherapy plays a role only in patients with lower tumor by improving the DFS. And in patients with upper tumor, the addition of adjuvant radiotherapy seemed unnecessary. The multivariable analysis further confirmed that adjuvant radiotherapy was independent prognostic factor for DFS, LRFS and DMFS in pT3N0 rectal cancer with lower tumor.

Many studies suggested additional radiotherapy in patients with T3 N0 rectal cancer was unnecessary due to the low local recurrence rate after an TME surgery. One study performed by Merchant NB [13] reported that the overall local recurrence was 9% and overall survival was 75% for pT3N0 rectal cancer patients who underwent surgery without adjuvant treatment. Similarly, in the study of Nissan et al., a low local recurrence rate of 4.1% was even reported in pT3N0 patients who received TME surgery alone [3]. Considering the excellent local control in pT3N0 rectal cancer after TME surgery, several researchers questioned the clinical value of adjuvant radiotherapy in these patients [7, 8]. In the study of Kim et al., 151 patients with stage IIA rectal cancer who received adjuvant chemotherapy (CT) (*n* = 29) or concurrent chemo-radiotherapy(CRT) (*n* = 122) following TME were enrolled. After a median follow-up period of 78 months, no significant differences were observed in the 5-year local recurrence or 5-year overall survival rates between CT and CRT, indicating additional postoperative radiotherapy did not alter local recurrence or survival in patients with stage IIA rectal cancer after TME [8]. In another study done by Park et al., 390 patients with stage IIA were enrolled. Adjuvant chemotherapy was provided to 180 patients (46.2%), and chemotherapy plus radiotherapy was provided to 210 patients (53.8%). Results showed that local recurrence rate did not differ between patients who did and did not receive radiotherapy [7]. Furthermore, in patients with mid and lower rectal cancer, the local recurrence rate was not affected by radiotherapy [7, 14], which was contradicted to our findings. Possible explanation was that we chose the cut-off of 7 cm from AV to separate the patients into lower and upper cancer groups while Park et al. used the cut-off of 5 and 10 cm to divide the patients into three groups. In our study, we also found that upper rectal cancers had a lower recurrence rates than mid and lower rectal cancers, though the difference was not significant (data not presented). Besides, Gunderson et al. found that pT3N0 rectal cancer acquired similar prognosis with T1/2 N1 rectal cancer after surgery plus chemotherapy, with the 5-year OS and DFS of 84 and 69%, respectively. And the adding of radiotherapy did not improve the survival [15].

The highlight in our work was that we performed further analysis based on clinical factor of tumor location. And we found that tumor location can serve as an indication for the use of adjuvant radiotherapy in pT3N0 rectal cancer after surgery, which has not been clearly suggested in current clinical practice. As we know, adjuvant radiotherapy can cause some toxicities which affect the life quality of rectal cancer patients who received adjuvant radiotherapy. The acute toxicities after postoperative radiotherapy occurred in 4 to 48% of cases, and serious toxicities requiring hospitalization or surgical intervention occurred in 3 to 10% of cases [16]. Besides, patients who did receive radiotherapy tended to suffer more radiation associated morbidities and dysfunctional outcomes such as fibrosis, autonomic nerve injury, and sexual dysfunction, compared to those who did not [17–19].

There were some risk factors which were found to be associated with the local recurrence. For example, abnormal CEA level, presence of LVI and perirectal fat invasion were all risking factors for local failure in pT3N0 rectal cancer. Additionally, Tepper et al. found that the number of lymph nodes harvested and the lower rectal cancer also predicted local recurrence [20]. Our findings were in consistent with the previous studies, especially the one performed by Tepper et al. We demonstrated that pre-treatment CEA level, tumor location and number of retrieved lymph node were independent predictors of LRFS. Specifically, upper rectal cancer was found to suffer lower local recurrence rates than lower rectal cancer [21–23]. Thus, we further performed subgroup analysis based on tumor location, finding that tumor location can be an indication for the administration of adjuvant radiotherapy in pT3N0 rectal cancer after surgery. We also found that tumor volume can predict DFS, which was not reported previously.

There were several limitations regarding our study. This was a retrospective analysis and the sample size in subgroup was relatively small. Although we tried to identify the value of adjuvant radiotherapy for pT3N0 rectal cancer, this question can only been answered by the large prospective clinical trial. Besides, the regimens of adjuvant chemotherapy given to the patients varied from each other, which may affect the results in some extent.

## Conclusions

In conclusion, adjuvant radiotherapy improved the DFS in pT3N0 rectal cancer patients with lower tumor. However, it showed no effect in patients with upper tumor. Tumor location can be an indication for the use of adjuvant radiotherapy in pT3N0 rectal cancer after surgery.

## Abbreviations

CI: Confidence interval
CRM: Circumferential resection margin
CRT: Concurrent chemo-radiation
CT: Chemotherapy
CTV: Clinical target volume
DFS: Disease-free survival
DFS: Disease-free survival
DMFS: Distant metastasis-free survival
LR: Local recurrence
LRFS: Local recurrence-free survival
NA: Not available
OS: Overall survival
SM: Systemic metastases
TME: Total mesorectal excision

## References

[1] Kachnic LA, Hong TS, Ryan DP (2008). Rectal Cancer at the crossroads: the dilemma of clinically staged T3, N0, M0 disease. J Clin Oncol.

[2] Bokey EL, Ojerskog B, Chapuis PH, Dent OF, Newland RC, Sinclair G (1999). Local recurrence after curative excision of the rectum for cancer without adjuvant therapy: role of total anatomical dissection. Br J Surg.

[3] Nissan A, Stojadinovic A, Shia J, Hoos A, Guillem JG, Klimstra D, Cohen AM, Minsky BD, Paty PB, Wong WD (2006). Predictors of recurrence in patients with T2 and early T3, N0 adenocarcinoma of the rectum treated by surgery alone. J Clin Oncol.

[4] Sauer R (2002). Adjuvant and neoadjuvant radiotherapy and concurrent Radiochemotherapy for rectal Cancer. Pathol Oncol Res.

[5] Vonk DT, Hazard LJ (2010). Do all locally advanced rectal cancers require radiation? A review of literature in the modern era. J Gastrointest Oncol.

[6] Enker WE, Thaler HT, Cranor ML, Polyak T (1995). Total mesorectal excision in the operative treatment of carcinoma of the rectum. J Am Coll Surg.

[7] Park IJ, Kim HC, Yu CS, Kim TW, Jang SJ, Kim JC (2008). Effect of adjuvant radiotherapy on local recurrence in stage II rectal cancer. Ann Surg Oncol.

[8] Kim JS, Kim NK, Min BS, Hur H, Ahn JB, Keum KC (2010). Adjuvant radiotherapy following total mesorectal excision for stage IIA rectal cancer: is it beneficial?. Int J Color Dis.

[9] Wo JY, Mamon HJ, Ryan DP, Hong TS (2011). T3N0 rectal Cancer: radiation for all?. Semin Radiat Oncol.

[10] Zoccali M, Fichera A (2012). Role of radiation in intermediate-risk rectal Cancer. Ann Surg Oncol.

[11] You KY, Huang R, Zhang LN (2015). Tailored selection of the interval between neoadjuvant chemoradiotherapy and surgery for locally advanced rectal cancer: analysis based on the pathologic stage or chemoradiation response. J Cancer Res Clin Oncol.

[12] Rengan R, Paty P, Wong WD (2005). Distal cT2N0 rectal cancer: is there an alternative to abdominoperineal resection?. J Clin Oncol.

[13] Merchant NB, Guillem JG, Paty PB, Enker WE, Minsky BD, Quan SH, Wong D, Cohen AM (1999). T3N0 rectal cancer: results following sharp mesorectal excision and no adjuvant therapy. J Gastrointest Surg.

[14] Wu JX, Wang Y, Chen N (2014). In the era of total mesorectal excision: adjuvant radiotherapy may be unnecessary for pT3N0 rectal cancer. Radiat Oncol.

[15] Gunderson LL, Sargent DJ, Tepper JE, Wolmark N, O'Connell MJ, Begovic M, Allmer C, Colangelo L, Smalley SR, Haller DG, Martenson JA, Mayer RJ, Rich TA, Ajani JA, MacDonald JS, Willett CG, Goldberg RM (2004). Impact of T and N stage and treatment on survival and relapse in adjuvant rectal cancer: a pooled analysis. J Clin Oncol.

[16] Ooi BS, Tjandra JJ, Green MD (1999). Morbidities of adjuvant chemotherapy and radiotherapy for resectable rectal cancer: an overview. Dis Colon Rectum.

[17] Glimelius B, Grönberg H, Järhult J, Wallgren A, Cavallin-Ståhl E (2003). A systematic overview of radiation therapy effects in rectal cancer. Acta Oncol.

[18] Minsky BD, Conti JA, Huang Y, Knopf K (1995). Relationship of acute gastrointestinal toxicity and the volume of irradiated small bowel in patients receiving combined modality therapy for rectal cancer. J Clin Oncol.

[19] Shibata D, Guillem JG, Lanouette N, Paty P, Minsky B, Harrison L, Wong WD, Cohen A (2000). Functional and quality-of-life outcomes in patients with rectal cancer after combined modality therapy, intraoperative radiation therapy, and sphincter preservation. Dis Colon Rectum.

[20] Tepper JE, O'Connell M, Niedzwiecki D, Hollis DR, Benson AB, Cummings B, Gunderson LL, Macdonald JS, Martenson JA, Mayer RJ (2002). Adjuvant therapy in rectal cancer: analysis of stage, sex, and local control–final report of intergroup 0114. J Clin Oncol.

[21] Folkesson J, Birgisson H, Pahlman L, Cedermark B, Glimelius B, Gunnarsson U (2005). Swedish rectal Cancer trial: long lasting benefits from radiotherapy on survival and local recurrence rate. J Clin Oncol.

[22] Faerden AE, Naimy N, Wiik P, Reiertsen O, Weyessa S, Trønnes S, Andersen SN, Bakka A (2005). Total mesorectal excision for rectal cancer. Difference in outcome for low and high rectal cancer. Dis Colon Rectum.

[23] Lee SH, de Hernandez Anda E, Finne CO, Madoff RD, Garcia-Aguilar J (2005). The effect of circumferential tumor location in clinical outcomes of rectal cancer patients treated with total mesorectal excision. Dis Colon Rectum.

